# Effects of Heating Mode and Temperature on the Microstructures, Electrical and Optical Properties of Molybdenum Thin Films

**DOI:** 10.3390/ma11091634

**Published:** 2018-09-06

**Authors:** Haili Zhao, Jingpei Xie, Aixia Mao, Aiqin Wang, Yanfang Chen, Tingting Liang, Douqin Ma

**Affiliations:** 1 School of Physical Engineering, Zhengzhou University, Zhengzhou 450052, China; hlzhao921@126.com (H.Z.); max0906@126.com (A.M.); 2 School of Materials Science and Engineering, Henan University of Science and Technology, Luoyang 471023, China; aiqin_wang888@163.com (A.W.); chenyanfang-78@163.com (Y.C.); tingting_liang520@163.com (T.L.); 3 School of Physical and Engineering, Henan University of Science and Technology, Luoyang 471023, China; 4 Collaborative Innovation Center of Nonferrous Metals, Henan University of Science and Technology, Luoyang 471003, China; madouqin1987@163.com

**Keywords:** CIGS thin film solar cells, Mo thin films, magnetron sputtering, heating mode, temperature

## Abstract

In this paper, molybdenum (Mo) thin films are deposited on soda-lime glass (SLG) substrates by direct current magnetron sputtering and heated in three different modes at different temperatures, including substrate heating, annealing treatment, and both substrate heating and annealing treatment. The effects of heating temperature and heating mode on the structures, morphology, optical and electrical properties of Mo thin films were systematically investigated by X-ray diffraction (XRD), Scanning electron microscopy (SEM), atomic force microscope (AFM) and UV-visible spectrophotometer (UV-vis spectra). It is shown that as the substrate and annealing temperature increase, the crystallinity of Mo thin films is improved, and the grain sizes become bigger. Especially in the mode of both substrate heating and annealing treatment at higher temperature, the obtained Mo thin films show higher crystallinity and conductivity. Moreover, with the increase of substrate and annealing temperature in different heating modes, both the surface compactness of Mo films and the optical reflectance increase correspondingly. Furthermore, the Mo film, prepared at the substrate heating temperature of 400 °C and annealed at 400 °C, showed excellent comprehensive performance, and the resistivity is as low as 1.36 × 10^−5^ Ω·cm. Using this optimized Mo thin film as an electrode, copper indium gallium selenium (CIGS) solar cells have a maximum photo-conversion efficiency of 12.8%.

## 1. Introduction

Molybdenum (Mo) thin film obtained by sputtering method has been the primary choice as the back contact material for thin film solar cells, such as the Cu(In,Ga)Se_2_ (CIGS)-based solar cell. Specifically, this is a copper indium gallium selenide thin-film solar cell (or CIGS cell, sometimes CI(G)S or CIS cell) used to convert sunlight into electric power. It is manufactured by depositing a thin layer of copper, indium, gallium and selenide on glass or plastic backing, along with electrodes on the front and back to collect current. Thermal and chemical stability during high temperature deposition, outstanding electrical conductivity, excellent adhesion on glass, and formation of ohmic contact with CIGS via an unintentionally induced adventitious p-MoSe_2_ interfacial layer have rendered Mo more advantageous than other metals [1,2]. Additionally, Mo thin films can also affect the out-diffusion of sodium from soda lime glass (SLG) substrates [3,4,5]. These make Mo a primary choice as a back-contact material, not only for the common CIGS material, but also for the industry standard for novel and selenium-free absorber layers, such as Cu_2_ZnSnS_4_ (CZTS), CuSnS_3_ (CTS) and SnS [6,7,8]. The properties and microstructures of Mo thin films could be controlled and adjusted to promote the functionality of solar cells in industry [9] and their morphology can be obviously changed from dense to porous, depending on the processing parameters, such as the sputtering pressure, sputtering power, substrate temperature, annealing temperature, substrate-target distance and type of discharge [10,11,12,13]. These in turn affect the residual stress, conductivity, optical reflectance and adhesion with soda-lime glass (SLG) [14]. Furthermore, because Mo back electrode materials need to withstand a certain high temperature during the preparation of solar cells, it is particularly important to study the characteristics of Mo thin films prepared under high temperature. In particular, the effects of the substrate heating and annealing process on CIGS-based solar cells have attracted considerable attention. Substrate heating and annealing can promote crystal growth and affect the properties of Mo thin film. However, to the best our knowledge, the effects of two heating modes and heating temperature on the microstructures and properties of Mo film have never been compared and investigated systematically.

Herein, the effects of heating mode and temperature on the microstructures and properties of molybdenum thin films were investigated in detail. Mo thin films were deposited by the direct current (DC) magnetron sputtering method at different substrate temperatures, and then annealed at different temperatures under a high-purity Argon gas atmosphere. Specifically, three experimental processes were designed as follows: (i) the effects of substrate temperature on the microstructures and properties of deposited Mo thin films were investigated; (ii) for Mo thin films deposited at room temperature, the effects of different annealing temperature on the microstructures and photoelectric properties of Mo films were studied; (iii) the films deposited at different substrate temperatures were annealed at different temperatures and the effects of annealing temperature and substrate temperature on the microstructures and properties of Mo films were studied. In order to quantitatively analyze the effects of substrate and annealing temperature on the crystal structure and surface morphology evolution of Mo films, Mo films were probed and compared using various methods that would affect the performance of the corresponding solar cell device. Using the optimized Mo thin films as electrodes, the highest photo-conversion efficiency of 12.8% is achieved for the CIGS thin-film solar cells.

## 2. Materials and Methods

In this work, the preparation of Mo thin films includes two processes: DC magnetron sputtering at different substrate temperatures and then subsequent vacuum annealing at different temperatures in a tube furnace. Mo thin films were deposited on SLG substrates with the size of 20 mm × 20 mm × 1 mm. Uniform films were achieved using a rotatable substrate holder (10 rpm) with a target—substrate distance of 8 cm away from the fixed circular target, which has a high purity of 99.97% and a diameter of 50 mm. Prior to sputtering, SLG substrates were washed using acetone, ethanol and deionized water for 15 min, respectively. After that, they were dried by N_2_ and then placed in the deposition chamber at once. The base pressure in the chamber was controlled below 2.0 × 10^−4^ Pa. Prior to each deposition, the pre-sputtering process was maintained for 15 min to remove impurities from the target surface so as to ensure the cleanliness of the target. In all of the following experiments, the sputtering power and pressure were kept as 100 W and 0.3 Pa, respectively. Both sputtering time and annealing time are set to 0.5 h. To research the effects of substrate and annealing temperature on the microstructures and properties of the as-prepared Mo thin films, three experimental procedures were designed: (i) Mo thin films were deposited by DC magnetron sputtering at five different substrate temperatures of 100 °C, 200 °C, 300 °C, 350 °C and 400 °C, respectively; (ii) the films were deposited by DC magnetron sputtering at room temperature and then were annealed at 100 °C, 200 °C, 300 °C, 350 °C and 400 °C, respectively; (iii) the films were deposited by DC magnetron sputtering at 100 °C, 200 °C, 300 °C, 350 °C and 400 °C, respectively, and then annealed at 300 °C and 400 °C, respectively. In order to avoid their oxidation, all samples were cooled down naturally after the sputtering and annealing.

CIGS absorbers with a thickness of about 1.5 μm were deposited on the sputtered Mo electrode/SLG substrates via a 3-stage co-evaporation process [15]. CdS buffer layers (~50 nm) was prepared by chemical bath deposition (CBD), intrinsic zinc oxide (i-ZnO) (~80 nm)/n-type indium tin oxide (ITO) (~250 nm) window layers were obtained through sputtering method and 500 nm thick aluminum (Al) grid electrode were evaporated. The CIGS thin film solar cells with the structure of glass/Mo/CIGS absorber/CdS/i-ZnO/n-ITO/Al were then fabricated and no anti-reflection coating was employed in current case.

The effects of different heating modes and temperature on the crystal properties of Mo thin films were recorded by X-ray diffraction (XRD, Panalytical, Almelo, Netherlands) using Cu Kα radiation (0.15406 nm) in 2*θ* scanning mode, and the step size was 0.02°. Scanning electron microscopy (SEM, ZEISS, Oberkochen, Germany) at an operating voltage of 6 kV was operated to characterize the morphologies of sputtered Mo thin films. An atomic force microscope (AFM, SPA-400, Tokyo, Japan) working in tapping mode with a scan speed of 3 Hz and a scan area of 5 μm was used to determine the surface roughness of the films. The reflectance was measured by UV-visible spectrophotometer (Hitachi U-4100, Tokyo, Japan) in the wavelength range of 300–900 nm. The degree of adhesion was qualitatively tested using a Scotch tape test. The electrical parameters, such as carrier concentration and mobility, were measured by the Hall effect measurement system, HMS ECOPIA 3000, with a magnetic field of 0.57 T and probe current of 10 mA for all the samples. The energy conversion efficiency was measured and calculated using the current–voltage (I–V) characteristics for the CIGS, under standard AM 1.5 and 100 mW/cm^2^ illumination at 25 °C. The average grain size (*D*) of the films was calculated using the Scherrer formula [16]:

(1) $$\;D=\frac{0.9\lambda }{\beta cos\theta }\;$$

where *λ* is the X-ray wavelength (0.15406 nm) and β is the full width at half maximum (FWHM) of the film diffraction peak at 2*θ* in radians—FWHM is an expression of the extent of function given by the difference between the two extreme values of the independent variable at which the dependent variable is equal to half of its maximum value. *θ* is the Bragg’s diffraction angle in degrees. The micros-train, *ε*, and dislocation density, ρ, developed in the thin films are calculated from Equations (2) and (3), respectively [17,18]:

(2) $$\;\varepsilon =\frac{\beta }{4tan\theta }\;$$

(3) $$\;\rho =\frac{n}{D^{2}}\;$$

where *n* is a factor which is almost equal to unity for minimum dislocation density and *D* is the grain size.

## 3. Results and Discussion

### 3.1. The Effects of Heating Mode and Temperature on Crystal Structures of Films

**Figure 1:**
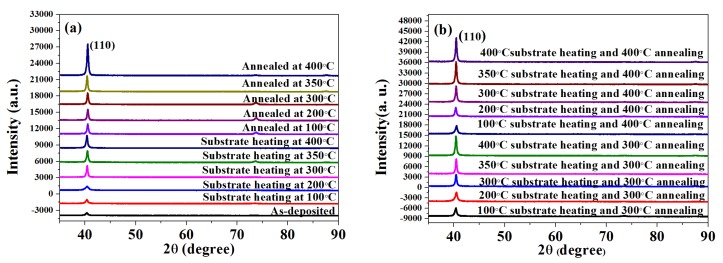
X-ray diffraction patterns of the Mo films heated (**a**) at various substrate temperatures and annealing temperatures and (**b**) at various substrate temperatures, then annealed at 300 °C and 400 °C.

Figure 1 shows the XRD patterns of Mo films deposited on SLG substrates at various substrate temperatures and annealing temperatures in different heating modes. All the films grow along the (110) plane, which is typical for Mo films with a body centered cubic (bcc) structure (JCPDS Card No. 3-065-7442). This is because the (110) plane of the bcc phase generally has the lowest surface energy and thus tends to grow preferentially. This result indicates that all Mo films synthesized at various substrate and annealing temperature have bcc structures.

The variation of (110) peak intensity, grain size, lattice parameters and strain values of the Mo thin films heated in different heating modes at different temperatures are shown in Table 1. It can be seen that the (110) peak intensity of the annealing samples is stronger than that of substrate heating at the same temperature, and the grain sizes of the annealing samples are larger. Furthermore, the Mo films prepared by the substrate heating and annealing have the strongest (110) peak intensity and the largest average grain size at the same temperature in different heating modes, and the higher the temperature is, the stronger the (110) peak intensity and the larger average grain sizes the Mo films have as the substrate and annealing temperature increase from 100 °C to 400 °C. The increase of (110) peak intensity and average grain size indicate that increasing the substrate and annealing temperature from room temperature (RT) to 400 °C enhances the crystallinity of Mo thin films. This may be due to the fact that a higher substrate and annealing temperature provide higher energy to the sputtered particles and then enhance surface mobility and diffusibility, facilitating their nucleation and growth on the substrate. Thus, they can fill up the microvoids and/or vacancies, resulting in better crystallinity and enhancing the particle growth [19]. The results are consistent with the conclusions in the literature [20]. On the other hand, as the substrate and annealing temperature increase, the densities of the crystallographic defects in Mo thin films also decrease rapidly, including dislocations, interstitials and vacancies [21].

It is worth discussing that the peak position shifts as the heating modes and temperature change. In other words, the lattice parameter of the deposit, and hence strain, within the films can be changed obviously in the different heating modes and temperature. The exacted position and characteristics of each peak were determined by fitting the data using a pseudo-Voigt function [22]. From the (110) peak position, the lattice parameter of the Mo bcc structure can be calculated and the relative variation compared to the Mo reference lattice parameter (a_0_ = 3.1473 Å) displays the extent of strain in the crystal lattice [23]. Negative or positive values can be deduced, which represent compressive or tensile strain, respectively.

As shown in Table 1, the calculated lattice parameters of the Mo films range from 3.1592 Å to 3.1430 Å. The results indicate that as the substrate and annealing temperature increase, the obtained tensile strain decreases slightly, while at higher temperatures, they become compressive strain (Table 1). This change is attributed to the voids, crystal impurities, oxygen or argon impurities which are responsible for the strain in the prepared Mo films, because these effects are related to the energy of Mo atoms [24,25,26]. At higher substrate and annealing temperatures, the sputtered Mo atoms have higher energy to enhance their surface mobility and then fill up microvoids and/or vacancies. This results in better crystallinity, larger grain size, fewer voids and a lower inclusion of impurities (i.e., Ar or O) in Mo thin films. Thus, at higher substrate and annealing temperatures, the as-obtained films have higher conductivity and less tensile stress. However, as the strain is further reduced at very high temperatures, it becomes compressive strain because the surface mobility of sputtered Mo atoms exceeds the intra-grain tensile forces.

### 3.2. The Effects of Heating Modes and Temperature on the Morphologies of Mo Thin Films

**Figure 2:**
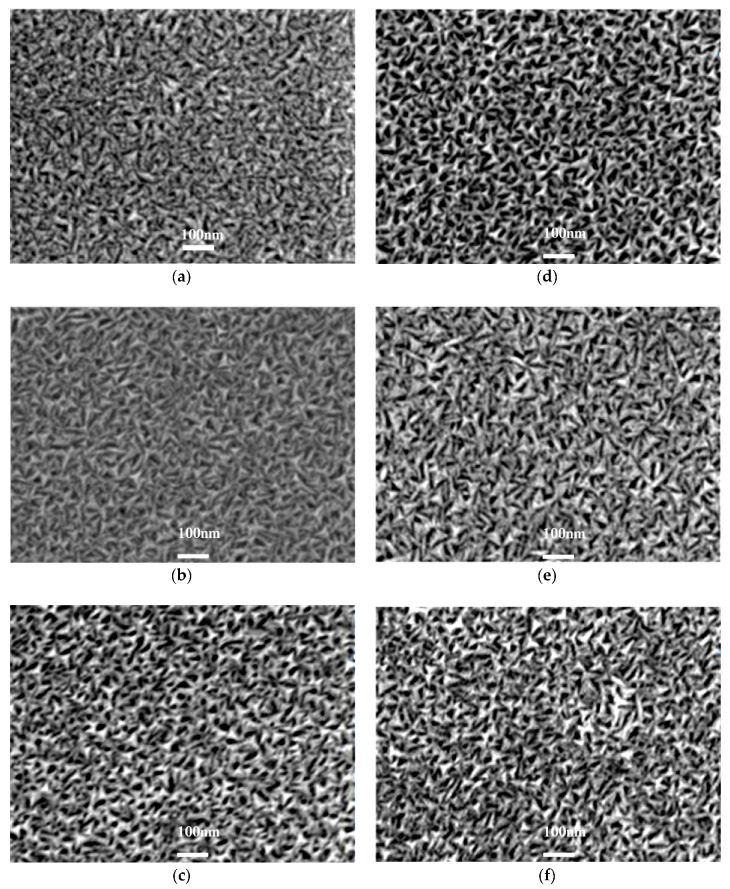
SEM images of Mo films deposited at different substrate temperature—(**a**) RT; (**b**) 200 °C; and (**c**) 400 °C—and at various annealing temperatures—(**d**) 100 °C; (**e**) 200 °C; (**f**) 400 °C, respectively.

Figure 2 shows the SEM images of Mo thin films prepared in different heating modes at different temperatures. It can be seen that all the films consist of triangular nanoparticles with uniform size distribution. With the increase of substrate and annealing temperature, the average grain size of Mo thin films increases slightly. At the same temperature, the average grain size of the annealing Mo thin films is slightly larger than that of the heating ones in substrate heating mode. This may originate from the fact that, at the same temperature, the annealed Mo films undergo a secondary crystallization process. Thus, the Mo particles absorb more energy and the grains grow more easily, leading to better crystallinity and larger grain sizes. Furthermore, Mo thin films prepared at higher temperatures exhibit a rougher surface. This can be determined by AFM and the root mean square (RMS) roughness of the as-prepared Mo thin films, as listed in Table 2. Specifically, regarding the Mo thin films deposited in substrate heating mode, the RMS roughness gradually increases from 2.36 nm to 5.62 nm when the substrate temperature is increased from 100 °C to 400 °C. Then, in annealing mode, the RMS roughness gradually increases from 3.63 nm to 6.35 nm when the annealing temperature is increased from 100 °C to 400 °C. The increase of RMS values is due to the coarse grains. Higher surface roughness can be expected for bigger grain clusters due to the bigger height differences between particular grains’ peaks and bases [27].

**Figure 3:**
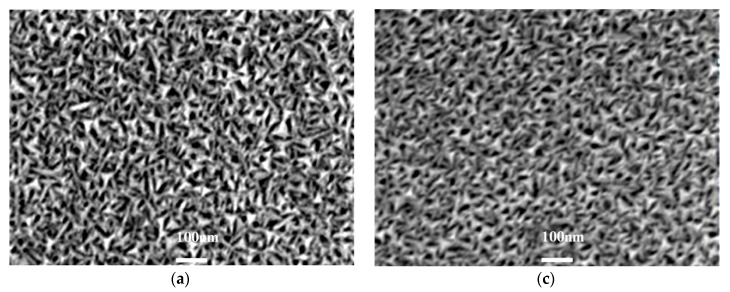

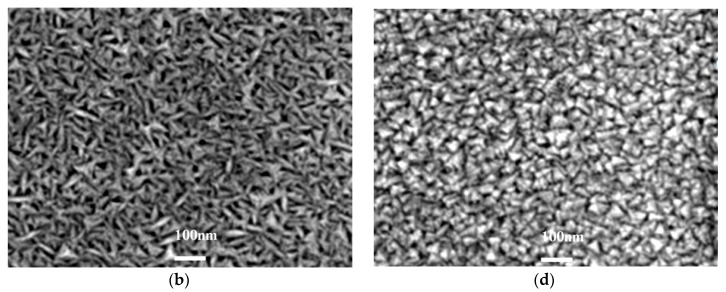
SEM images of Mo films deposited at different substrate temperature and annealing temperatures: (**a**) 200 °C substrate heating and 300 °C annealing; (**b**) 400 °C substrate heating and 300 °C annealing; (**c**) 200 °C substrate heating and 400 °C annealing; and (**d**) 400 °C substrate heating and 400 °C annealing.

Figure 3 shows SEM images of Mo thin films prepared in both substrate heating and annealing mode at different temperatures, from which similar results can be obtained. That is, the grain size of Mo films increases with the increase of substrate and annealing temperature and all the films are composed of triangular nanoparticles. At 300 °C and 400 °C annealing temperature, the RMS surface roughness of Mo thin films deposited at different substrate temperature increases from 2.36 nm to 4.85 nm and from 3.48 nm to 6.35 nm, respectively (see Table 2). The films prepared with higher substrate and annealing temperature show a rougher surface. Similar to the previous analysis, at a higher temperature, the nanoparticles in Mo films absorb more energy, leading to stronger surface mobility and diffusiatbility. Thus, they have more opportunities to merge around the particles and grow, and thus larger nanoparticles are formed.

In addition, for the Mo thin films fabricated at the same substrate temperature, the grain sizes via annealing at 400 °C are bigger than those at 300 °C. The reason for this is that the energy of Mo particles at 400 °C annealing temperature is higher than that of 300 °C. Thus, the RMS roughness is also higher at an improved annealing temperature.

### 3.3. The Effects of Heating Mode and Temperature on Electrical Properties

**Figure 4:**
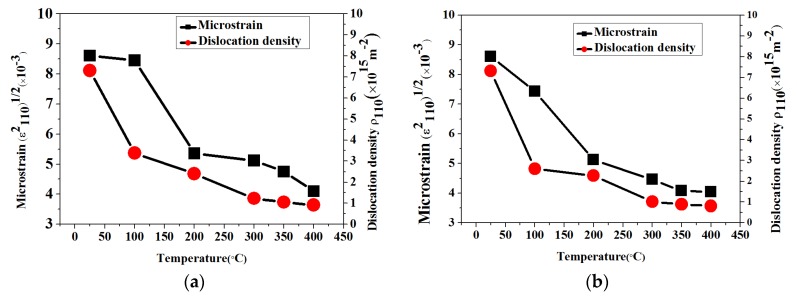

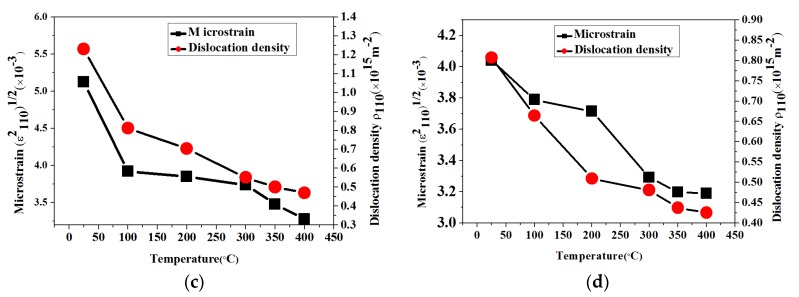
Microstrain and dislocation density variations of Mo thin films prepared in different heating modes at different temperature: (**a**) substrate heating; (**b**) annealing; (**c**) both substrate heating and 300 °C annealing; and (**d**) both substrate heating and 400 °C annealing.

The average microstrain (*ε*) and the dislocation density (ρ) are shown in Figure 4. It can be seen that in different heating modes, both microstrain and dislocation density decrease with the increased substrate and annealing temperature, which can directly decrease the resistivity of Mo thin films. Brown researched the effect of dislocations on conductivity and proved that electron mobility was inversely proportional to the density of randomly distributed dislocations [28]. Consequently, the resistivity was shown to be proportional to the dislocation density. This is due to the fact that the more energy the Mo films absorb, the stronger mobility and diffusibility the nanoparticles in Mo films have, the better the crystallinity Mo films exhibit, and the less dislocation and microstrain Mo films have. Therefore, as the carrier mobility and carrier concentration increase, the resistivity decreases with the decrease of microstrain and dislocation density. In fact, the resistivity is caused by the mean squared microstrain, and there is a linear relationship between microstrain and dislocation density [29,30,31]. Thus, the resistivity shows a linear decrease trend with the decrease of dislocation density [32].

The electrical properties of Mo thin films are also summarized and shown in Table 2. As the substrate and annealing temperature increase from 100 °C to 400 °C for Mo thin films prepared in substrate heating mode, the resistivity decreases from about 3.50 × 10^−5^ Ω·cm to 2.50 × 10^−5^ Ω·cm, and for the as-deposited Mo thin films annealed at different temperatures, the resistivity decreases from about 3.50 × 10^−5^ Ω·cm to 2.32 × 10^−5^ Ω·cm. For the Mo thin films prepared at different substrate temperature and 300 °C annealing temperature, the resistivity decreases from about 2.32 × 10^−5^ Ω·cm to 1.52 × 10^−5^ Ω·cm, and at 400 °C annealing temperature, the resistivity decreases from about 1.99 × 10^−5^ Ω·cm to 1.36 × 10^−5^ Ω·cm.

Electron conduction is dependent on the electron scattering process caused by structural imperfections including grain boundaries, dislocation, impurities, microstrain and point defects. In general, the increase of grain size leads to a decrease trend in grain boundaries (GBs) in a certain amount per volume, which charge carriers have to cross during the electrical transport [33]. Grain boundaries are 2D defects in the crystal structure and tend to block the carrier transition and increase the resistivity. At the same time, the grain boundaries in the materials are often considered as the source of dislocations. Consequently, when the substrate and annealing temperature increase from 100 °C to 400 °C, the grain sizes increase, the number of grain boundaries decrease, and the dislocation density decreases. Apart from the increase of grain size, the randomly distributed dislocation core, which causes the resonance scattering of Fermi electrons, also has a big influence on the resistivity variation in films through carrier mobility limitation. In all samples, the Mo thin film prepared at 400 °C substrate heating and 400 °C annealing temperature has the biggest grain size, the least grain boundaries, the weakest electron scattering, and the smallest resistivity.

### 3.4. The Effects of Heating Modes and Temperature on Optical Properties of Mo Thin Films

**Figure 5:**
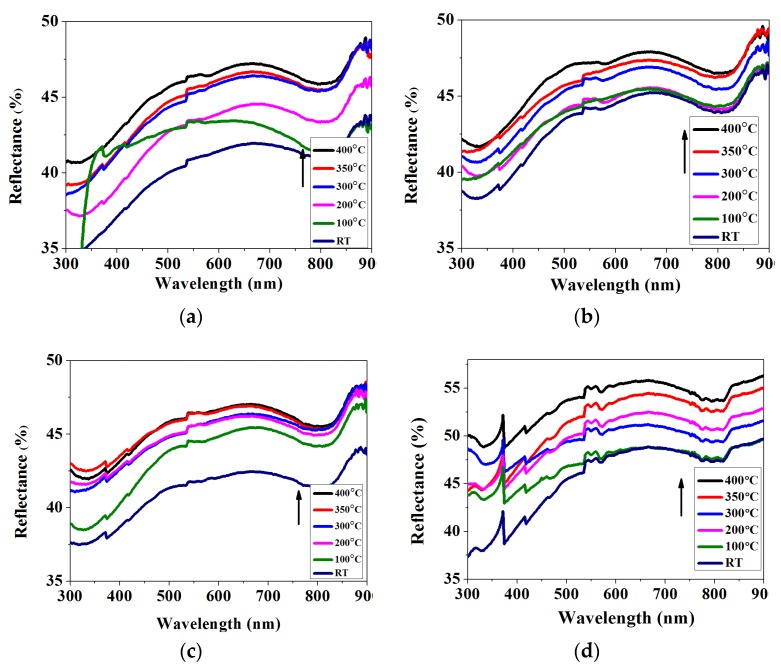
Reflectance of Mo thin films deposited at (**a**) different substrate temperatures; (**b**) different annealing temperatures; (**c**) different substrate temperatures and 300 °C annealing temperature; and (**d**) different substrate temperatures and 400 °C annealing, respectively.

The reflectance of Mo films is also crucial to solar cell efficiency, because the optimization of cells’ back reflectance can give non-absorbed light a second chance to be harvested by the active cell layer, so higher reflectance can improve their efficiency [34]. Figure 5 shows the reflectance of Mo thin films prepared in different heating modes. Mo thin films prepared at higher substrate temperature and annealing temperature exhibit higher reflectance. The reflectance increases with the increasing substrate temperature and annealing temperature. At 400 °C substrate temperature and 400 °C annealing temperature, it reaches a maximum.

This dramatic increase in reflectance can be mainly attributed to the increase of compactness and grain size, which result in the decrease of scattering of light. It is also found that the grain sizes of Mo thin films, prepared in different heating modes at different temperatures, increase from 11.7 to 48.5 nm and their structures become more compact compared to the samples prepared at lower substrate and annealing temperatures. This is because that the higher annealing and substrate temperature can provide the Mo particles with sufficient free energy to enhance surface migration, grain growth and grain aggregates. Then, the reconstruction of the crystals and positive changes in the optical properties of the films take place. As Mo grains grow, the crystal surface energy is released and the defects in the film are reduced accordingly. At the same time, with the increasing substrate temperature and annealing temperature, the surface areas of films become smaller, which reduce the stress in the films. Therefore, the heating treatment under higher substrate and annealing temperature has the ability to increase the film grain size and reduce their stress for improving the reflectivity [35].

### 3.5. The Effects of Heating Modes and Temperature on CIGS Solar Cell

**Figure 6:**
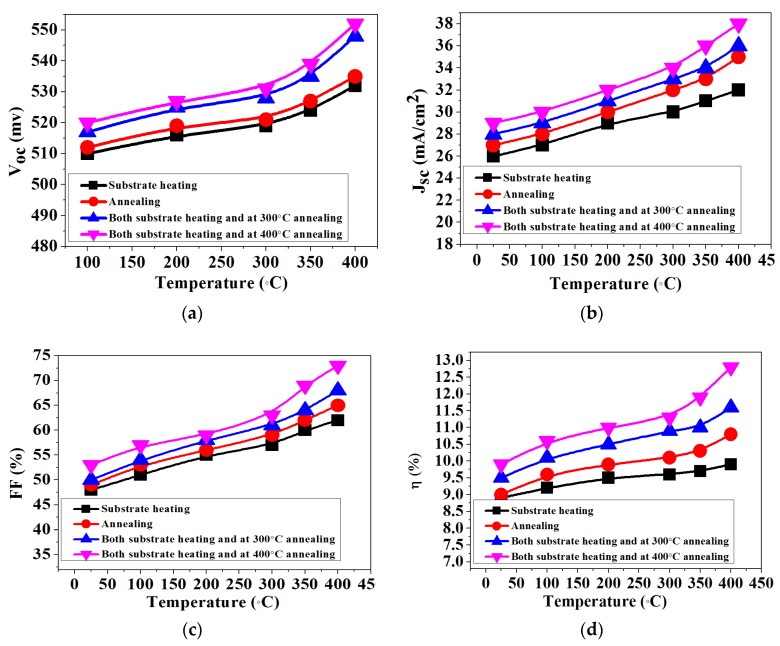
I–V parameters of copper indium gallium selenium (CIGS) thin-film solar cells as a function of temperature of Mo electrodes heated in three different modes: (**a**) change of the open circuit voltage (Voc) with different temperature; (**b**) change of the short circuit current density (Jsc) with different temperature; (**c**) change of the fill factor (FF) with different temperature; and (**d**) change of the photo-conversion efficiency(η) with different temperature.

The CIGS thin film solar cells with the glass/Mo/CIGS absorber/CdS/i-ZnO/n-ITO/Al grid structures are fabricated on the Mo electrodes prepared in different heating modes at different temperature. Figure 6 shows the current–voltage (I–V) parameters as a function of heating modes and temperature of Mo electrodes. The results show that the open circuit voltage (Voc) increases with the increase of temperature in different heating mode for all CIGS thin films solar cells (Figure 6a). With the decrease of resistivity caused by the increasing substrate and annealing temperature of sputtered Mo electrodes, the fill factor (FF) of CIGS thin films increases. The short circuit current density (Jsc) for the CIGS solar cell, mainly coming from the light absorption, also increases with the increase of substrate and annealing temperature. The photo-conversion efficiency of the CIGS solar cells changes in the range of 8.9–12.8%. Furthermore, the Mo film prepared at 400 °C substrate heating and 400 °C annealing temperature shows excellent comprehensive performance: its resistivity reaches to 1.36 × 10^−5^ Ω·cm and the reflectance is above 50%. Using this optimized Mo thin film as an electrode, a maximum photo-conversion efficiency of 12.8% is achieved for the copper indium gallium selenium (CIGS) solar cells.

## 4. Conclusions

In summary, Mo thin films are prepared on soda-lime glass substrates by DC magnetron sputtering and heating in different modes at different temperatures, including substrate heating, annealing treatment, and both substrate heating and annealing treatment. It is found that the heating modes and temperature had remarkable influences on the structures and properties of the as-prepared Mo thin films. As the substrate and annealing temperature increase, the crystallinity of Mo thin films is improved, and the grain sizes become bigger. The crystallinity of the annealing samples is better than that of substrate heating at the same temperature, and the grain sizes of the annealing samples are larger. Furthermore, the Mo films prepared by the substrate heating and annealing have the best crystallinity and the largest average grain size at the same temperature in different heating modes, and the higher the temperature is, the better the crystallinity and the larger the average grain sizes the Mo films have as the substrate and annealing temperatures increase from 100 °C to 400 °C. Especially in the mode of substrate heating and annealing at higher temperatures, the as-prepared Mo thin films show higher crystallinity and conductivity. With the increase of substrate and annealing temperature in different heating modes, both the surface compactness of Mo films and the optical reflectance increase correspondingly. Furthermore, the Mo film prepared at 400 °C substrate heating and 400 °C annealing temperature exhibits excellent comprehensive performance, and its resistivity reaches to 1.36 × 10^−5^ Ω·cm, which is lower than the general resistivity reported in other literature. Using this Mo thin film as an electrode, the highest photo-conversion efficiency of 12.8% was achieved for the CIGS thin-film solar cells.

## Figures and Tables

**Table 1:** Adhesion, lattice parameter, grain size, intensity of (110) peak and strain of Mo thin films prepared in different heating modes at different temperature.

Sample Identification	Substrate Temperature (°C)	Post Annealing Temperature (°C)	Lattice Parameter (Å)	Grain Size (nm)	Intensity of (110) Peak	Strain (%)	Adhesion (Pass/Fail)
A-0	RT	---	3.1592	11.7	904	0.3813	Pass
A-1	100	---	3.1581	17.2	1210	0.3495	Pass
A-2	200	---	3.1570	20.4	1224	0.3178	Pass
A-3	300	---	3.1562	28.5	3403	0.2860	Pass
A-4	350	---	3.1553	30.9	3706	0.2542	Pass
A-5	400	---	3.1544	33.1	4569	0.2224	Pass
B-0	---	RT	3.1592	11.7	904	0.3813	Pass
B-1	---	100	3.1554	19.6	1332	0.2542	Pass
B-2	---	200	3.1531	21.0	1456	0.1907	Pass
B-3	---	300	3.1522	31.3	3608	0.1589	Pass
B-4	---	350	3.1483	33.6	4735	0.0318	Pass
B-5	---	400	3.1441	35.2	5062	−0.105	Pass
C-1	100	300	3.1531	35.1	3623	0.1907	Pass
C-2	200	300	3.1524	37.7	6501	0.1589	Pass
C-3	300	300	3.1512	42.6	6806	0.1271	Pass
C-4	350	300	3.1472	44.7	8215	0.0000	Pass
C-5	400	300	3.1434	46.1	8502	−0.127	Pass
D-1	100	400	3.1482	38.8	5831	0.0318	Pass
D-2	200	400	3.1451	44.3	6012	−0.064	Pass
D-3	300	400	3.1447	45.6	8326	−0.075	Pass
D-4	350	400	3.1446	47.8	8893	−0.076	Pass
D-5	400	400	3.1430	48.5	9229	−0.127	Pass

**Table 2:** The resistivity, grain size, Hall mobility, carrier concentration and RMS roughness of Mo thin films prepared in different heating modes at different temperatures.

Sample Identification	Grain Size (nm)	Resistivity (×10^−5^ Ω·cm)	Hall Mobility (cm^2^/Vs)	Carrier Concentration (10^22^ cm^−3^)	RMS Roughness (nm)
A-0	11.7	3.50	7.95	10.87	2.36
A-1	17.2	3.28	8.21	11.28	3.03
A-2	20.4	3.03	8.88	12.01	3.62
A-3	28.5	2.86	9.00	12.86	3.95
A-4	30.9	2.62	9.16	13.24	4.02
A-5	33.1	2.50	9.31	13.56	4.35
B-0	11.7	3.50	8.32	12.69	2.36
B-1	19.6	3.15	8.65	12.01	3.38
B-2	21.0	3.00	8.96	12.63	3.83
B-3	31.3	2.58	9.20	13.41	4.26
B-4	33.6	2.45	9.66	13.80	4.53
B-5	35.2	2.32	9.98	13.96	4.31
C-1	35.1	2.35	10.22	14.03	4.30
C-2	37.7	2.01	11.12	14.85	4.85
C-3	42.6	1.86	11.35	15.32	5.26
C-4	44.7	1.66	11.98	16.02	5.48
C-5	46.1	1.52	12.36	17.29	5.60
D-1	38.8	1.99	10.54	15.02	4.90
D-2	44.3	1.72	11.36	15.58	5.46
D-3	45.6	1.58	12.65	16.95	5.52
D-4	47.8	1.49	12.88	17.78	6.01
D-5	48.5	1.36	13.62	17.82	6.24
